# Evaluation on the automotive skill competency test through ‘discontinuity’ model and the competency test management of vocational education school in Central Java, Indonesia

**DOI:** 10.1016/j.heliyon.2022.e08872

**Published:** 2022-02-02

**Authors:** Abdurrahman, Parmin, Stefanus Muryanto

**Affiliations:** a Mechanical Engineering Department, Faculty of Techniques, Universitas Negeri Semarang, 50229 Gunungpati, Semarang, Indonesia; b Science Education Studies Program, Faculty of Mathematics and Natural Sciences, Universitas Negeri Semarang, 50229 Gunungpati, Semarang, Indonesia; c Chemical Engineering Department, UNTAG University in Semarang, 50235 Gajah Mungkur, Semarang, Indonesia

**Keywords:** Vocational education, Automotive, Competency, Discontinuity model, Competency test management

## Abstract

This article provides an alternative competency test model for vocational education schools in Central Java, Indonesia which particularly for automotive skill competency in order to replace the current competency test model that has been implemented for several decades which the author's concern about the students' accomplishment during the competency test, aimed to improve competency test results to increase the students' job opportunity in the labor market. Currently, the applied competency test model is the ‘Continuity’ model, where the students are given five tasks and should be finished within five hours with limited break time. This model tends to increase students' fatigue and stress levels. Consequently, the students lose their focus and concentration which adversely impacts their competency test results. In this study, the new competency test model namely the ‘Discontinuity’ competency test model was proposed aiming to overcome the issue of the ‘Continuity’ model. To research the effect of the ‘Discontinuity’ model implementation on the students' competency test results and vocational school competency test management, a study was done among 100 students and 50 teachers in 10 vocational education schools around Central Java, Indonesia. The results show that the ‘Discontinuity’ competency test model gives a significant improvement in the students' competency test scores. This reasonably happened due to the implementation of the ‘Discontinuity’ model gives the students time to break for an hour of each competency task. Without this break time, fatigue and stress levels of the students will significantly increase which adversely affect the students' competency final score. In addition, the new management of the competency test was proposed in this research.

## 1 Introduction

The presence of vocational education in this present circumstance brings a significant impact on the correlation between education and the labor market. Students are given two choices whether they will continue their studies to regular school or vocational education. Vocational education becomes a good choice for students who wants to directly look for a job after graduating from school and could be a shortcut to being involved in the labor market directly. The percentage of practical knowledge of vocational education is higher than that in regular school. Thus, vocational education aims to prepare students one step forward for entering the labor market with a highly skilled in a specific field of jobs. In general, there are three types of vocational education systems, which are school-based vocational education, dual apprenticeship, and informal training (Guo and Wang, 2020). In a developing country, school-based vocational education is more applicable than the other systems considering manpower inequality. Students that enroll in a vocational education school exhibit outstanding practical skills supported by their early interest in the profession (Quiroga-Garza et al., 2020). The vocational education school carried out the high-intensity practical study with a specific industrial-based curriculum and competency (Muja et al., 2019). However, in some developing countries, manpower inequality in terms of practical skills and knowledge becomes a critical issue (Forster and Bol, 2018; Maragkou, 2020; Mohapatra et al., 1992; Xie et al., 2020). Thus, the role of a formal curriculum and standardized competency is very important to bring manpower equality which means the students are eligible for the industrial and business company requirements after graduating from vocational education. Moreover, vocational education also brings a significant impact on the economic sector for the country by reducing the youth unemployment population (Choi et al., 2019; Nilsson, 2010). Previous studies have investigated the effect of vocational education school-leavers on the labor market. The studies showed that the educational programs that were strongly oriented towards vocational skills and knowledge for the students have a positive influence and better integration into the labor market (Bol and van de Werfhorst, 2011; Muja et al., 2019).

### 1.1 The importance of competency for vocational education

However, in order to prepare the students' capability during the transition from high school to the labor market, the improvement of students' competency should be highly considered. Competency is a combination of skills, behavior/attitude, and multiple knowledge that can be demonstrated by students where the skills, attitudes, and knowledge are obtained from the materials conceptualization that has been learned during the period of study. The level of vocational competency influences positively the chance of graduates of being matched to occupation with the specific educational domain. Furthermore, the application of on-the-job training will be much more efficient when implementing the generic competencies to adjust vocational competencies to the requirement of the job (Heijke et al., 2003). Due to the increase in the labor market qualification, the standard of student competence should be increased. This concern should be followed by the development of the students’ assessment. Several points should be considered in order to improve the competency, which are authentic assessment, an improvement in quality lab sheet, student competency standard system, specific scoring rubric, and feedback from the students regarding their work. Therefore, a valid, reliable, fair, and consistent quality assessment could be achieved (Rahman et al., 2014).

In this industrial era, developing country such as Indonesia tends to force the economic sector by improving the industrial sector through massive production and export activity (Hidayatno et al., 2019; Neilson et al., 2020). In order to ensure that high demand in the industrial sector, vocational education contributes to human resources development and minimizes the gap between the academic environment and industrial needs (Salleh et al., 2015). Therefore, it is critical to set up a good competency including skills and organizational knowledge for the graduates which means the graduates are ready for entering the workplace environment. To meet the industrial requirements, the graduates should be able to work effectively by combining the knowledge, skills, and other work-related capacities into specific competence needed (Loon and Bartram, 2007). This matter could be achieved by implementing the internship program during the period of study in a vocational education school that has a related industrial field (Ocampo et al., 2020).

Competency test has been commonly used in most of the vocational education in Central Java, Indonesia, especially in the automotive field to ensure the capability of the students to conduct vehicles reparation or maintenance. The specific tasks are given to the students such as engine tune-up, clutch overhaul, gearbox transmission overhaul, electrical body system, and starter system. Currently, these five tasks are implemented to the students by using the ‘Continuity’ model which means that the students should finish all the tasks continuously within 5 h. However, the ‘Continuity’ model tends to cause work fatigue during the competency test, while the work fatigue will decrease the student's concentration and focus which will significantly cause poor competency test results. Nurhayati et al. investigated the effect of the increase in the production time on the productivity achieved. During the investigation, muscle fatigue has occurred at a very high level of production time and the results showed that the productivity achieved are below the productivity target (Nurhayati et al., 2016). Moreover, previous studies have investigated work fatigue as a long-term sickness absence. Hence, more potential diseases such as muscular soreness, cough, headache, and many more can cause the worker unfit. In addition, lack of focus and concentration in the workplace due to work fatigue means a lack of safety awareness which can cause fatal injury and death (Banks et al., 2019; Janssen, 2003). Based on the previous survey for the ‘Continuity’ test model showed unsatisfied results, whereas the students feel under pressure during the five hours competency test and under the assessor's supervision. Moreover, most of the students suffer from pressure which is not only physical but also mentally pressured. This condition caused the students' competency score results are not optimal. This will decrease the students' opportunity to get their best results in the competency test, while the competency test score determines their opportunity for competing in the labor market. Therefore, further evaluation of the ‘Continuity’ competency test model in vocational education schools is highly necessary for this global industrial era.

### 1.2 Management of the competency test

In vocational education, competency turns into an important aspect for the graduates as a benchmark that should be achieved by the students during their period of study to get a better job opportunity in the labor market. Competency has two essential values which are first, authorities in carrying out the responsibility, license or right to decide, produce, serve, act, and perform, and second, the capability to implement the knowledge, skills, and experience (Mulder, 2007). Competency tests in vocational education schools should have a comprehensive and multi-dimensional construction. Generally, there are three different competency levels, those are conceptual competence, procedural competence, and interpretative competence (Winther and Achtenhagen, 2009; Winther and Klotz, 2013). All these competency levels lead to job assignments that match the specific needs of the world business industry. Competency test is essential for the students in terms of the final decision whether the students pass or fail during their study in vocational education as well as to avoid the incompetent graduates in the labor market (Johnson, 2008). Alternatively, the substance of the competency test is not only for testing the students but also to seek the validation of the students. The validation process itself focuses on how students work on each assignment such as the used tools' accuracy, their work attitude, and adherence to SOP, instead of the final results-oriented only (pass or fail).

However, in this current vocational education school in Central Java, Indonesia, the implemented competency test is only focused on the final result without considering the process and performance of the students in detail during the competency test. The competency test is conducted only one time simultaneously at the end of the student's period of study according to the curriculum order. This management of the competency test is considered less effective because the implementation of the competency test is limited at a certain time. The impact adversely students' performance during the competency test as well as the assessors' validation of the students' performance. In addition, owing to the last semester's implementation of the competency test, the students tend to struggle to do retests if they fail during the competency test due to the limited schedule. Therefore, innovation in the management of the competency test is highly necessary in order to significantly improve the quality of vocational education graduates. Proper management of the competency test is proposed in this research which aimed to give the students a bigger opportunity to perform better and get their best result during the competency test. The proper management of the competency test allows the students to take the assignments at any time as long as the quota meets the minimum requirements. The school will provide a minimum quota for each task to be carried out. Thus, the assessor could give detailed assessment and validation based on the requested ongoing assignment whether the students are passed or failed on their competency test. However, if the student failed, they will be given a chance to practice more in the workshop during their study, then they could take another competency test in the next semester. The proposed management of the competency test is involving teachers, internal assessors, and external assessors (field experts: mechanics or automotive experts).

### 1.3 The effect of fatigue and stress level

The high intensity of the workload in the industrial sector tends to produce high fatigue and stress levels of the worker. This concept is also similar to the competency test particularly in automotive skills for vocational education students. During the automotive skill competency test, the students were forced to finish heavy duty within a limited time. However, this will significantly produce a poor competency result which will decrease the opportunity of the students entering the labor market due to the students’ fatigue and stress level increase. The study about the effect of fatigue and stress levels on productivity has been investigated over the decades. The results prove that the environment and work pressure significantly affect the body temperature of the workers whereas significantly affecting muscle fatigue and stress (Chad and Brown, 1995). Qualitatively, fatigue and stress levels are attributed to extended working hours, working conditions, and high workloads. These aspects are significantly affecting low productivity and low job satisfaction (Pelders and Nelson, 2019). Several impacts on the fatigue and stress level increased were observed such as reduced levels of awareness, low concentration and focus, reduced motivation and impaired mood as well as low job satisfaction (Lerman et al., 2012; Phillips et al., 2017; Shen et al., 2006). In terms of health, fatigue, and stress level can also lead to long-term health problems such as muscular tension, musculoskeletal disorders, heart disease, and mental illness which the workers tend to use sick leave and reduce productivity (Åkerstedt et al., 2014). However, recommendations such as time management, working time arrangement, rest and break time optimization, fitness, and sports program are highly necessary in order to avoid workers' fatigue and stress level increases (Hsouna et al., 2019; Safitri and Rusdiana, 2010).

Looking into the importance of rest and break time management and the time arrangement in the industrial sector, vocational education schools should implement a similar method due to vocational education graduates are focused on the industrial labor market (Ahmed, 2016; Pema and Mehay, 2012). The implementation of the fatigue and stress level management could be assigned to the students' competency test model. However, most vocational education schools in Indonesia are facing poor fatigue and stress management on their students during competency tests where poor competency results are inevitable (Abdurrahman et al., 2019). Meanwhile, the demand for the industrial sector on human resources is increasing with high criteria required. To overcome this problem, vocational education schools should increase their graduates’ competency test scores, thus their graduates could easily compete in the labor market and improve job opportunities.

### 1.4 The ‘discontinuity’ competency test model

Therefore, in this present study, the alternative model of competency test for vocational education called the ‘Discontinuity’ test model has been proposed and investigated in detail. The concept of the ‘Discontinuity’ model is by giving the student time to break and rest while shifting with another student to conduct the competency test. The five tasks of the competency test could be done within 10 h with a break time of 1 h in between each task. By giving the break time of 1 h, the competency test could be done by 10 students per day by doing the competency test alternately. The break time is very important and has been commonly used in the industrial sector to improve productivity and reduce the fatigue level of the workers (Gregson, 2020; Lim et al., 2020; O'Neill and Panuwatwanich, 2013). This current study aims to observe the student's convenience on the application of the ‘Continuity’ and ‘Discontinuity’ test model by analyzing the competency test score of the vocational education students in Central Java, Indonesia. This study is a development from the previous study which was conducted in a narrower area and the taken sample was only from one vocational education school in Semarang city, Indonesia (Abdurrahman et al., 2019). However, the developed ‘Discontinuity’ model in this study covers larger areas which are involving 10 vocational education schools in Central Java, Indonesia, and provides a better result for educational improvement.

## 2 Methods

**Figure 1:**
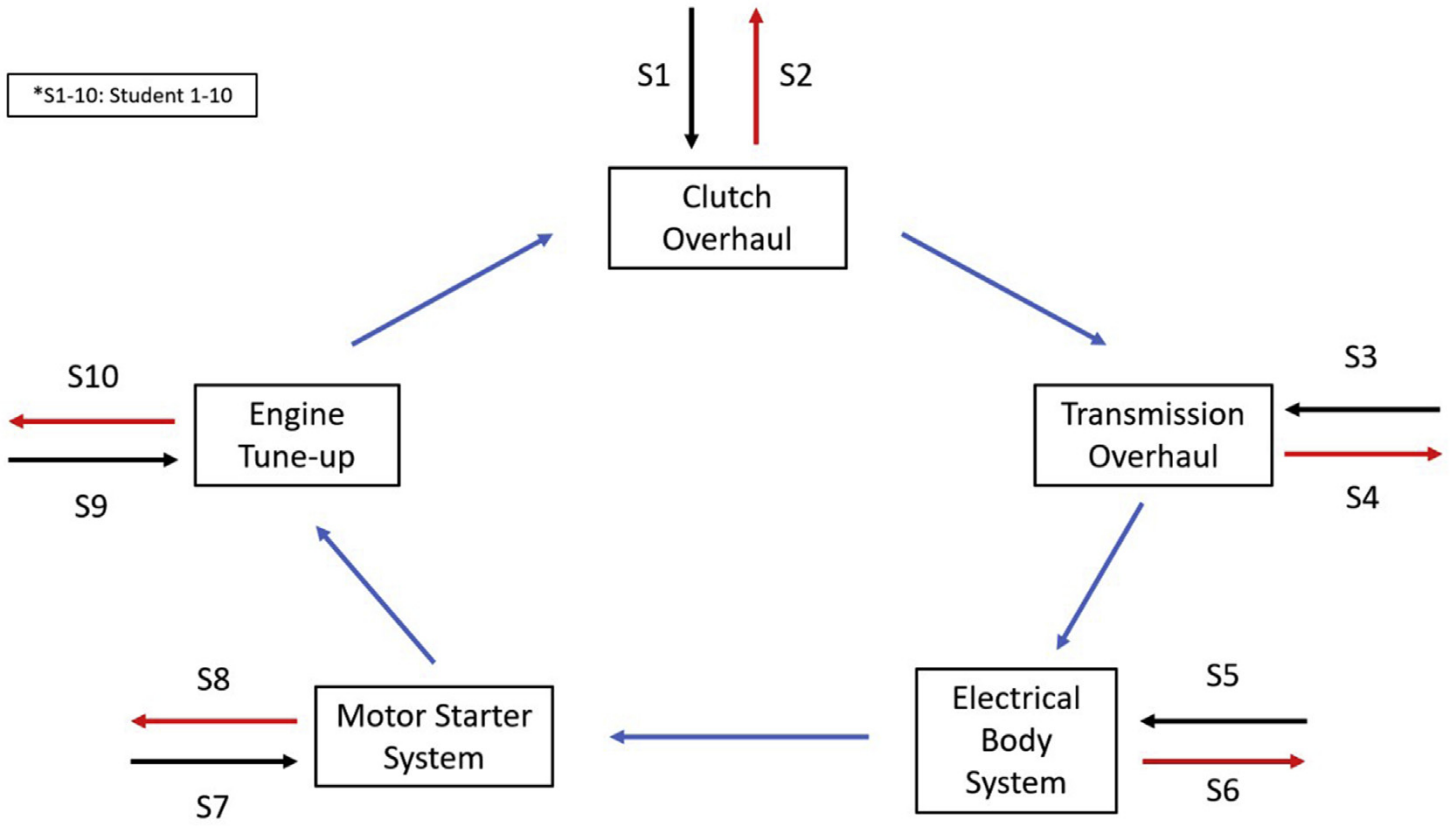
Schematic diagram of the ‘Discontinuity’ competency test model.

This study was focused on a descriptive quantitative method with data collected through a questionnaire that describes the educational reality regarding the level of student's satisfaction with vocational education in Central Java, Indonesia (Sánchez Prieto et al., 2020). The level of satisfaction in vocational education is determined by the ability of teachers in learning. This study also used a survey design because the number of research targets is spread over 10 different areas while the collecting data method is the same through competency tests and questionnaires. Two different models were implemented to collect the data which were ‘Continuity’ and ‘Discontinuity’ model competency tests of automotive skills in vocational education schools. The ‘Continuity’ model means the students must finish 5 competency tasks continuously within 5 h with a very limited time to break. Meanwhile, the ‘Discontinuity’ model means the students will be given a certain time (1 h) to break after finishing one task while shifting with the other student to do the same task. The schematic diagram of the ‘Discontinuity’ model is shown in Figure 1. The ‘Discontinuity’ model is considered to be more feasible and could significantly improve the students' competency test final score.

This study was conducted in Central Java, Indonesia which collecting data from 10 different Vocational High Schools (SMK) with the competence of the Automotive Light Vehicle Techniques which are; SMK Negeri 2 Surakarta, SMK Negeri 2 Salatiga, SMK Negeri 1 Magelang, SMK Negeri 1 SEDAN Rembang, SMK Negeri 4 Semarang, SMK Negeri 1 Kedungwuni, SMK Negeri 1 Ampelgading, SMK Negeri 2 Pati, SMK Negeri 2 Kudus, dan SMK Negeri 1 Adiwerna Tegal. Several stages have been carried out in this research. The following stages were explained as follows; determine the problem, determine the goal, make an instrument, determine the sample, collect data, analyze the data, conclude, and follow up the findings. In order to answer the aim of this study, three variables were implemented which are students’ fatigue during the competence test, students' score acquisition, and competency test management.

**Figure 2:**
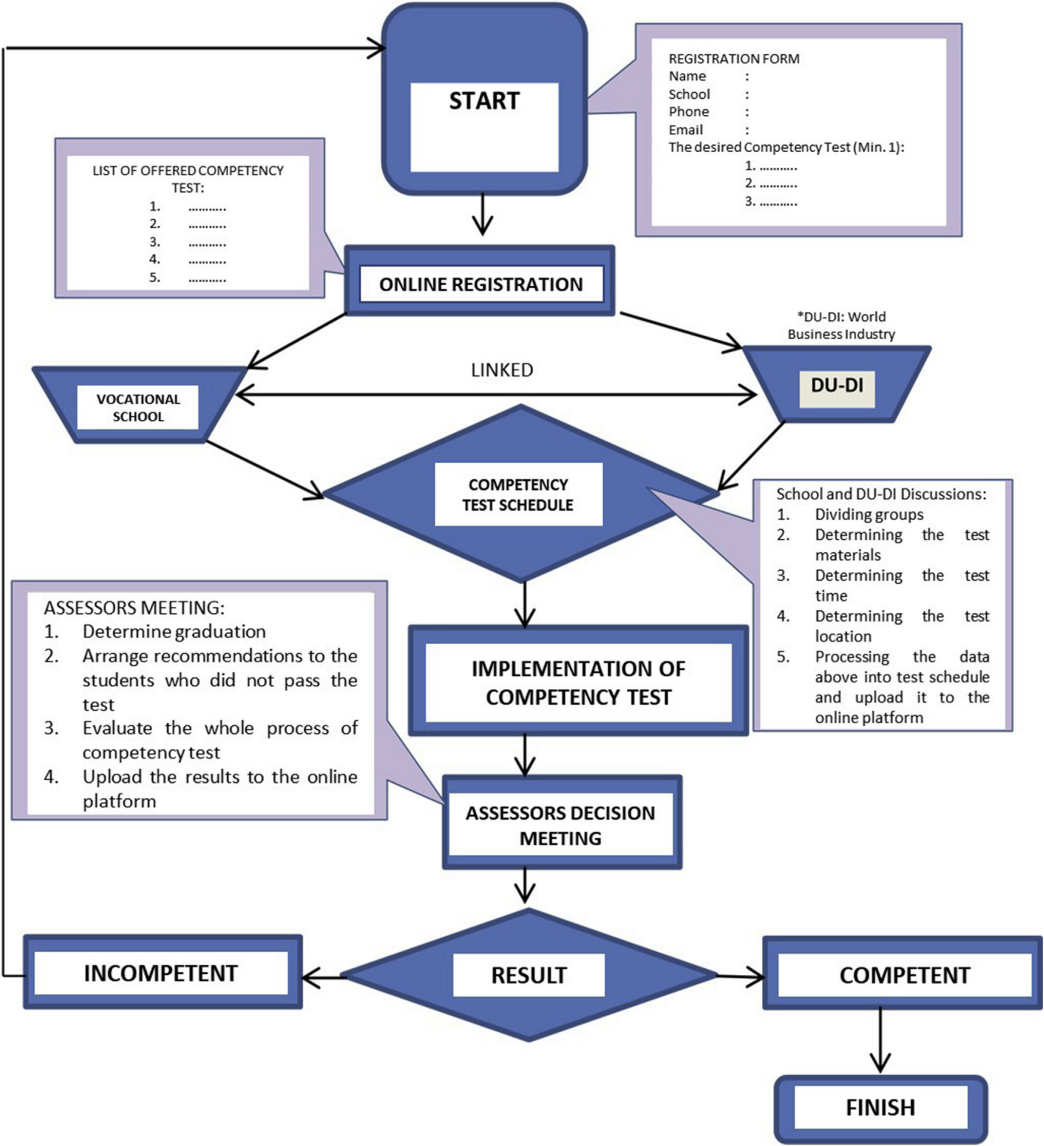
Schematic diagram of the proposed competency test management.

In terms of the proposed management of the competency test, the questionnaire was given to the respective teachers, head of the department, and head of the laboratory of each vocational school regarding their judgment of the proposed management to be implemented. The student's evaluation of the proposed competency test management should consist of the following skills, those are task skill, task management skill, contingency management skill, environment skill, and transfer skill. This proposed management will ease the students to get the optimum result of their competency test. The students are allowed to take the competency test at any time since the first semester of their study as long as meet the minimum requirements. The proposed competency test management could be accessed by the students through an online platform which is provided by the collaboration between the vocational schools and the world business industry. The schematic diagram of the proposed management of the competency test is shown in Figure 2.

The study uses descriptive research with a *sample at one point in time* model, which is a descriptive study that aims to report the characteristics of the object studied whose research data is obtained from one-time measurement only. Before using the instruments, the validity of the items for the research instrument was carried out by using the competency test for the teachers. The results of the validity of the item from 40 items were declared valid from 30 questions. There are 30 valid questions that are continued as a matter of competence test. There are 5 competency tests for students with 12 points each for the transmission overhaul, 10 points for the electrical body system, 8 points for the motor starter system, 14 items for the engine tune-up, and 8 points for the clutch overhaul. The samples consisted of 5 teachers from each school which has a total of 50 teachers and 10 students from each school which has a total of 100 students. The research data were collected through the application of the “Continuity” and “Discontinuity” competency test model. After the competency test, the questionnaires were given to the students to reveal the satisfactory level of using Continuity and Discontinuity models. In this study, a comparative test was conducted between continuity and discontinuity groups through T-Test analysis.

Moreover, related to the competency test management, the head of the department, head of the laboratory technician, and teachers were involved to observe and analyze the advantages and disadvantages of the proposed management given by the author. Additionally, the current study has been approved by the ethical committee of the “Indonesian Ministry of Education and Culture, Universitas Negeri Semarang, Lembaga Penelitian dan Pengabdian Masyarakat” and confirmed that the study complies with all regulations and confirmation that informed consent was obtained.

## 3 Results and discussion

**Figure 3:**
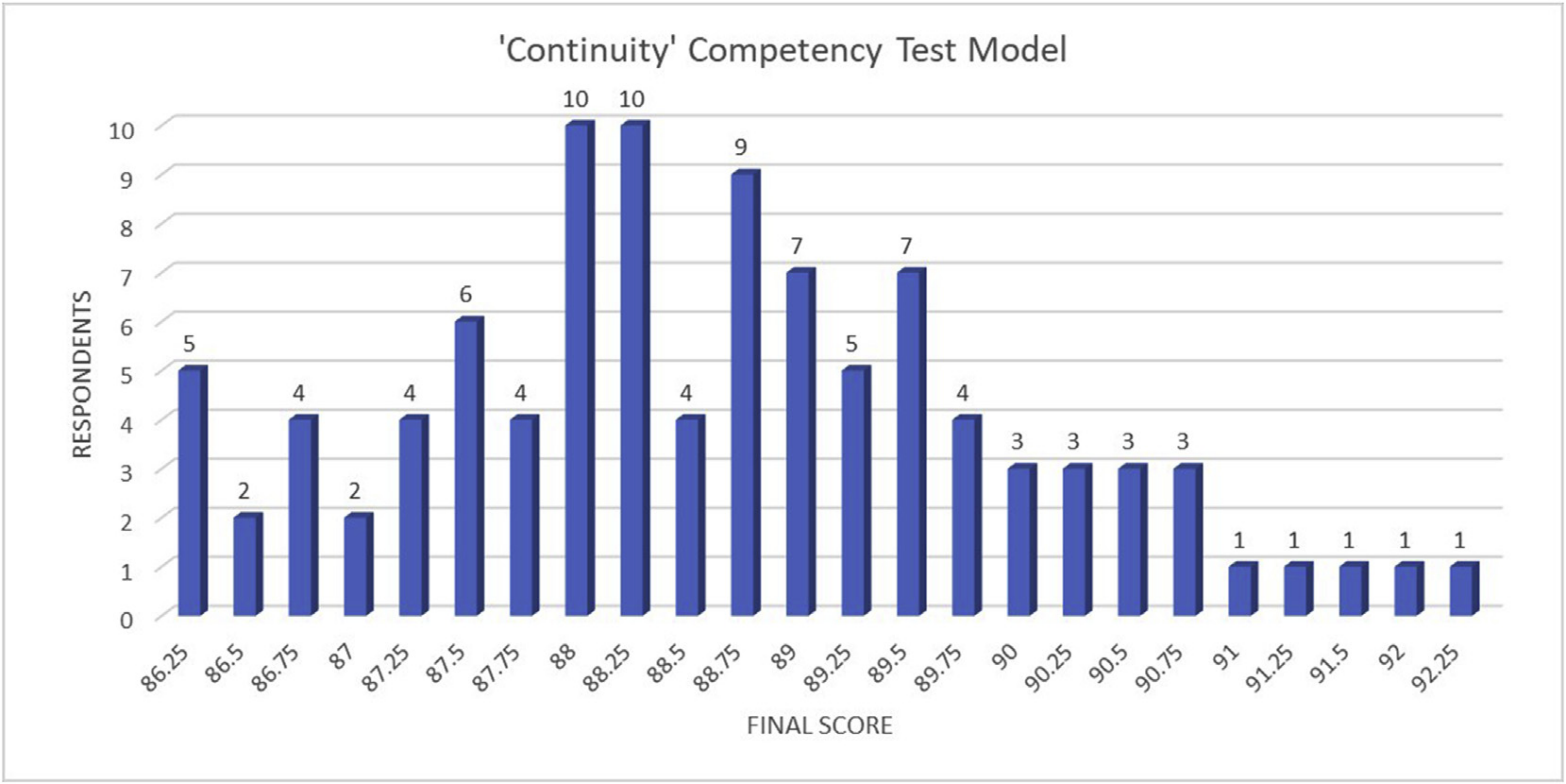
Schematic graph of the ‘Continuity’ competency test model results. Criteria: 50–60: Very low 61–70: Low 71–80: Enough 81–90: Good 91–100: Very good.

**Figure 4:**
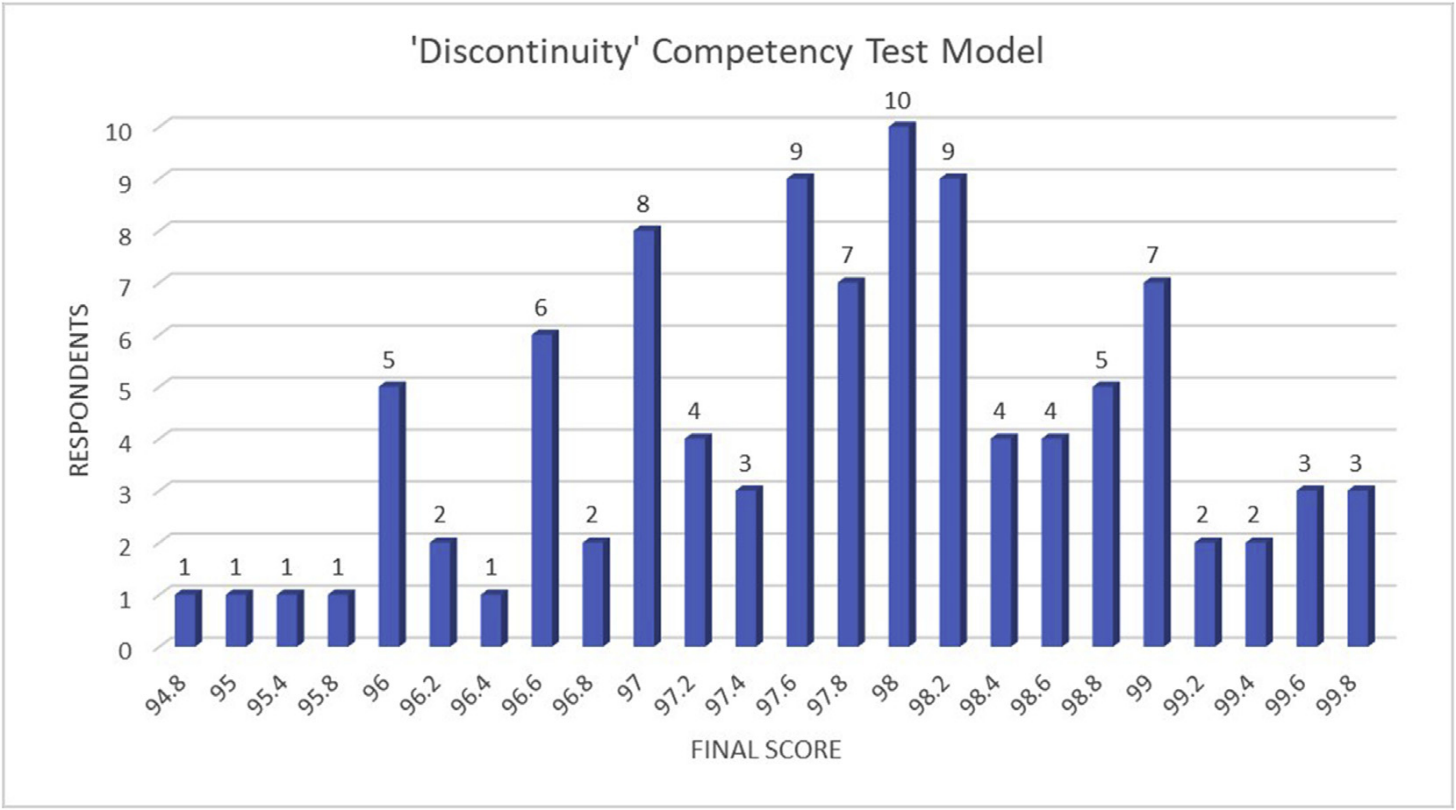
Schematic graph of the ‘Discontinuity’ competency test model results. Criteria: 50–60: Very low 61–70: Low 71–80: Enough 81–90: Good 91–100: Very good.

This present study revealed the comparison between the ‘Continuity’ test model and the ‘Discontinuity’ test model that has been done in vocational schools in Central Java, Indonesia within the period of March to April 2020. Figure 3 and Figure 4 indicate the correlation between the number of respondents and the final score of the competency test using the ‘Continuity’ and the ‘Discontinuity’ model, respectively.

Based on the ‘Continuity’ competency test model result which is shown in Figure 3 revealed that the final competency scores were mostly at the ‘Good’ level. The numbers of students with a ‘Good’ level were 86 students. Meanwhile, the students with ‘Very good’ level were only 14 students which are indicated in Table 1. The lowest score obtained by 5 students was 86.25 and 92.25 was the highest score for the ‘Continuity’ competency test model which was only achieved by one student. This score level indicates the students' capability and convenience during the competency test. In this global industrial era, a ‘very good’ level is highly necessary to be obtained by the students due to the high requirements of the manpower in these current industrial companies (Suharno et al., 2020). Thus, the improvement in the competency test results of the students should be carried out. Otherwise, vocational education schools fail to become a bridge between the graduates and the labor market.

**Table 1:** Students' competency score based on criteria classification of the ‘Continuity’ model.

Criteria	Very Low	Low	Enough	Good	Very Good
Total Students	-	-	-	86	14

Meanwhile, based on the ‘Discontinuity’ competency test model result which is shown in Figure 4 revealed that all of the final competency scores were at ‘Very good’ level. The lowest score was 94.8, while the highest score for the ‘Discontinuity’ competency test model was 99.8. Although both ‘Continuity’ and ‘Discontinuity’ competency test model results were still acceptable, the ‘Discontinuity’ competency test model emphasizes the optimum final score of the students. One hour of rest and break time that is given to the students during the competency test exhibit the optimum potential of the students. Thus, they could perform better during the competency test and deliver higher competency final scores that could be beneficial for their opportunity in the labor market. The result in Table 2 has also become evident that the students could achieve a “very good” result in their students' competency score using the discontinuity model.

**Table 2:** Students' competency score based on criteria classification of the ‘Discontinuity’ model.

Criteria	Very Low	Low	Enough	Good	Very Good
Total Students	-	-	-	-	100

**Figure 5:**
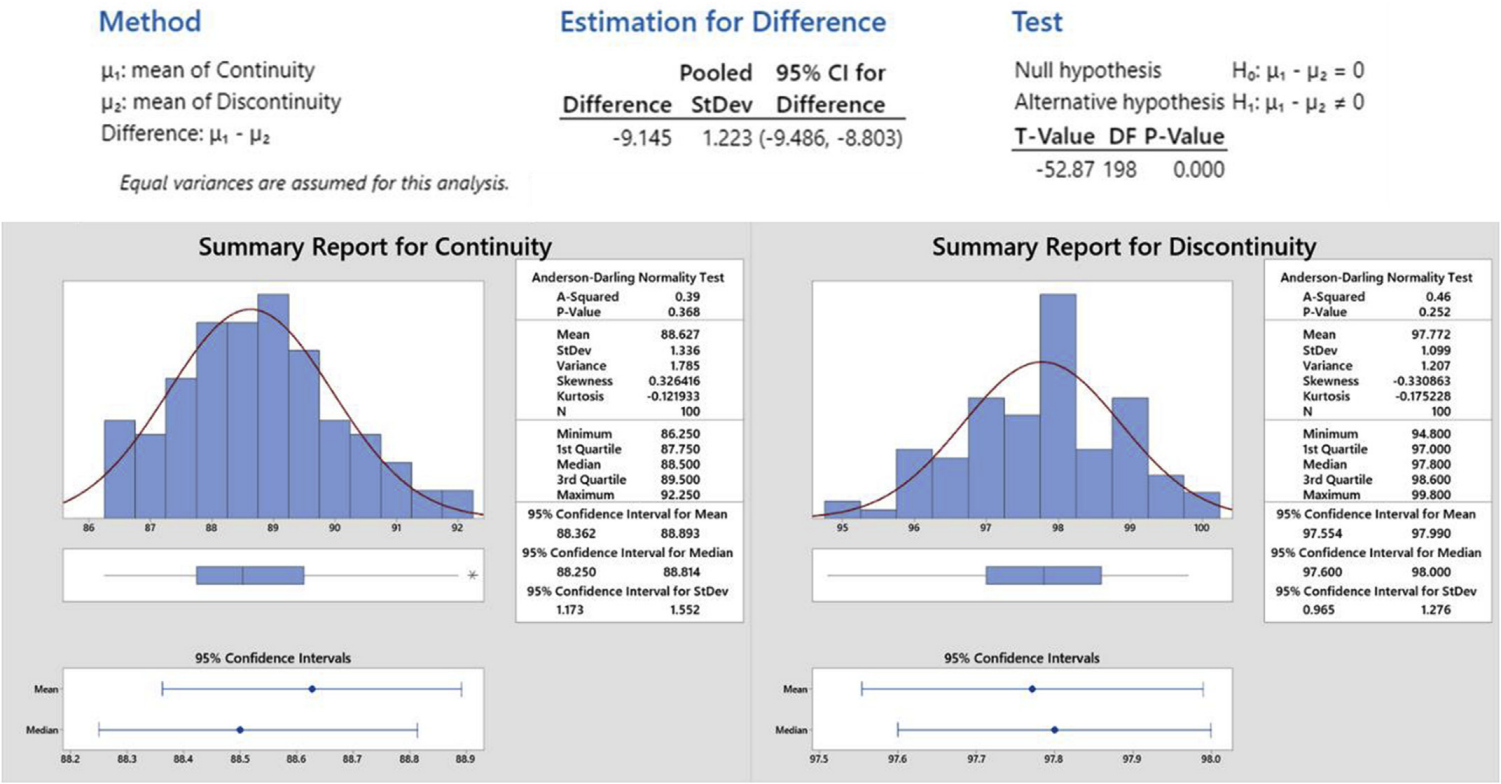
T-Test analysis result for the Continuity and Discontinuity model.

Sig = 0.000 = 0% < 5%, it means that Ho is rejected or H1 is accepted. The mean of continuity and discontinuity competency groups is different. Therefore, the mean of the discontinuity group is 97.77 which has a higher amount compared with the continuity group of 88.63 which is shown in Table 3. The results of the discontinuity competency group are better than the continuity competency group. Figure 5 also shows the summary report from the T-Test analysis of both the continuity and discontinuity model. The P-Value of discontinuity was 0.252 and the continuity was 0.368. It means that the lower P-value is better. According to the T-Test analysis, the discontinuity model has a better statistical model compared to the continuity model.

**Table 3:** Results of the T-Test in the continuity and discontinuity groups.

Group	N	Mean	Std. Deviation	Std. Error Mean
Continuity	100	88.6275	1.33593	0.133559
Discontinuity	100	97.7720	1.09877	0.10988

**Figure 6:**
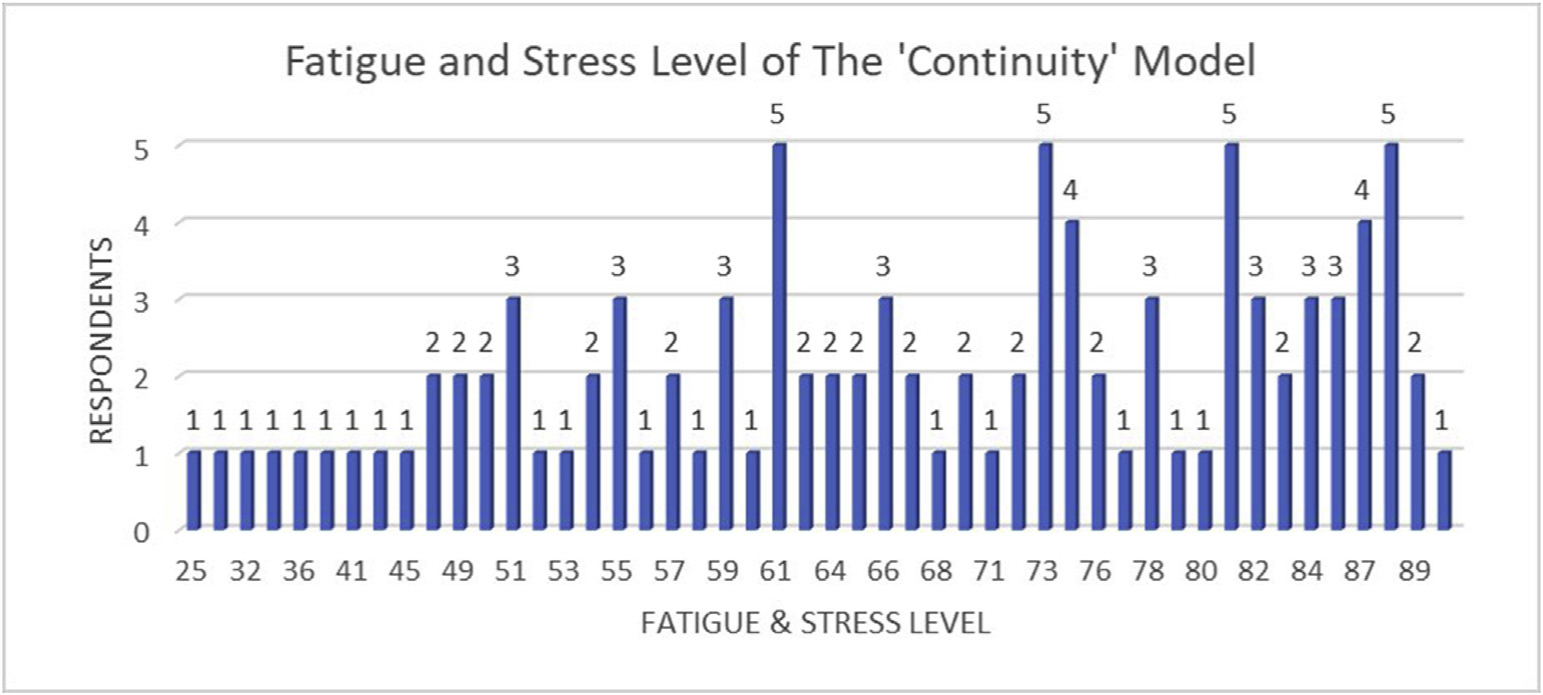
Schematic graph of the fatigue and stress level of the ‘Continuity’ competency test model. Criteria: 15–30: Very not tiring 31–45: Not tiring 46–60: Enough 61–75: Tiring 76–90: Very tiring.

Figure 6 and Table 4 indicated the fatigue level survey that has been given to the respondents regarding the ‘Continuity’ competency test model. The results revealed that students tend to feel very tiring when implementing this method. These results were in accordance with the final score of the competency test results which dominantly at the ‘Good’ level. Only one student stated in the ‘very not tiring’ category. Meanwhile, 24 students stated ‘enough’, 31 students stated ‘tiring’, and 36 students stated ‘very tiring’ category. These results were taken based on the students' experience during the implementation of the ‘Continuity’ competency test model. This result proves that the ‘Continuity’ competency test model significantly affects the students' fatigue and stress levels.

**Table 4:** Students' fatigue level criteria classification of the ‘Continuity’ model.

Criteria	Very Not Tiring	Not Tiring	Enough	Tiring	Very Tiring
Total Students	1	8	24	31	36

**Figure 7:**
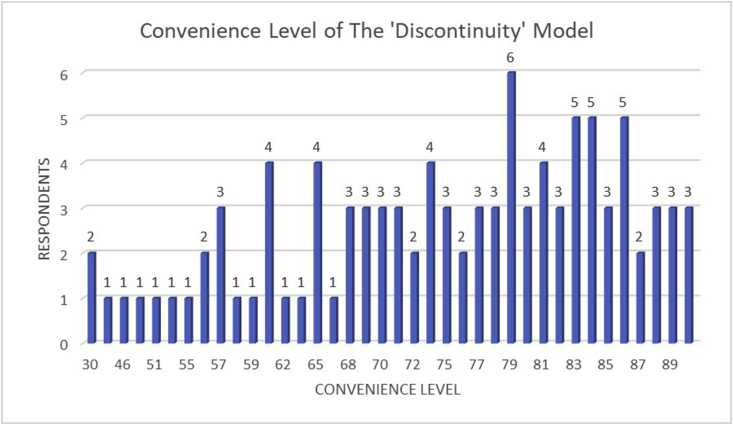
Schematic graph of the fatigue and stress level of the ‘Discontinuity’ competency test model. Criteria: 15–30: Very uncomfortable 31–45: Uncomfortable 46–60: Enough 61–75: Comfortable 76–90: Very comfortable.

Figure 7 and Table 5 revealed the survey result that has been given to the students according to their opinion on the implication of the ‘Discontinuity’ model regarding their convenience during the competency test. Based on the graph in Figure 6, it is clearly stated that most of the students are comfortable and very comfortable with the application of the ‘Discontinuity’ test model. It is in accordance with their final score of the competency test results which indicated significant improvement and better results during the competency test. 53 students stated in the ‘very comfortable’ category, 32 students stated in the ‘comfortable’ category, 12 students stated in the ‘enough’ category, 1 student stated in the ‘uncomfortable’ category, and only 2 students stated at ‘very uncomfortable’ category. This result proves that the application of the ‘Discontinuity’ test model gives better comfortability to the students as well as reduces their fatigue and stress level. Therefore, the final score of the competency test could be increased significantly. The ‘Discontinuity’ model gives the students the opportunity to take a break for an hour in between the tasks in order to recover their focus and concentration to conduct the next following competency test. Therefore, they can refresh their mind to avoid high-stress levels during competency tests and take a break to relax their body due to the high intensity and pressure of the competency test. This ‘Discontinuity’ competency test model is established to fulfill the students' desire to get the optimum competency test result to support their assets and increase their opportunities in the labor market. In terms of quality, the vocational education school in which implementing this method could significantly gain massive improvement by graduating students with a high competency level.

**Table 5:** Students' convenience level criteria classification of the ‘Discontinuity’ model.

Criteria	Very Uncomfortable	Uncomfortable	Enough	Comfortable	Very Comfortable
Total Students	2	1	12	32	53

**Figure 8:**
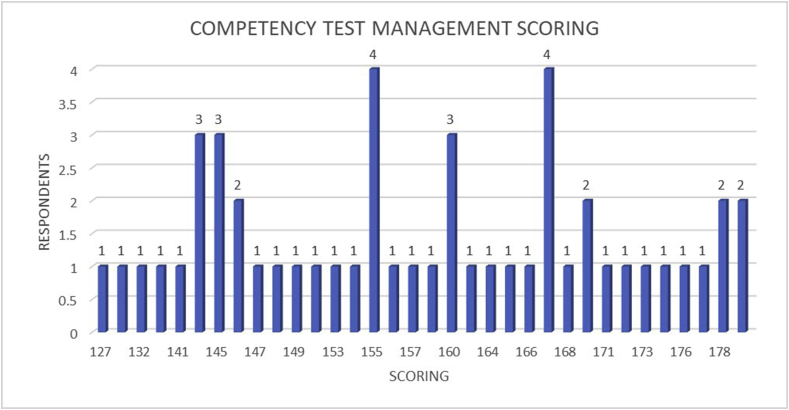
Schematic graph of the proposed competency test management scoring. Criteria: 30–60: Very disagree 61–90: Disagree 91–120: Enough 121–150: Agree 151–180: Very agree.

The questionnaire of feasibility scoring was given to the respective vocational education teachers and staff who have been involved in this research to evaluate the proposed competency test management. According to the feasibility scoring result of the proposed competency test management, all of the vocational education teachers were agree with the proposed competency test management which is shown in Figure 8 and Table 6 where 34 teachers stated ‘very agree’ and 16 teachers stated ‘agree’. This result proves that the benefits of the implication of the proposed competency test management are not only for the students but also for the teachers. Moreover, students' opportunities in the labor market improved owing to the students' competency final score improvement.

**Table 6:** Feasibility test result graph of the proposed competency test management.

Criteria	Very Disagree	Disagree	Enough	Agree	Very Agree
Total Respondents	-	-	-	16	34

## 4 Conclusion

Fatigue and stress levels of the vocational education students could adversely impact the competency test result. The current competency test model for vocational education in Central Java, Indonesia is a ‘Continuity’ competency test model. This model tends to give low scores on students' competency test results due to the lack of time to break where the students must finish five tasks within 5 h, thus, it causes the students' focus and concentration to decrease significantly. To overcome this problem, the implementation of the ‘Discontinuity’ model was proposed. By giving one-hour time to break for the student and the competency test could be done alternately, fatigue, and stress level of the students could significantly decrease. Consequently, the result shows that the final score of the student's competency test result was significantly improved. All the ‘Discontinuity’ model-based results showed in the ‘very good’ category. In addition, the survey results showed that the students feel more comfortable using the ‘Discontinuity’ competency test model than that ‘Continuity’ competency test model as well as the teachers satisfied with the proposed competency test model. The improvement in the competency test result is very important for the student who will be facing the labor market after graduating from vocational education. However, the industry considers students with a high competency score to join their company rather than the lower score one. In addition, a new competency test management is proposed in this study by allowing the students to take the competency test at any time since their first semester of study through an online platform that is directly connected with vocational school management and DU-DI (World Business Industry). The feasibility scoring survey of the proposed management revealed that both teachers and staff of the respective vocational schools in Central Java, Indonesia agree to implement the proposed competency test management. However, the proposed competency test management will ease the students and teachers to achieve an optimum final score of the competency test as well as improve the vocational school quality.

## Declarations

### Author contribution statement

Abdurrahman: Conceived and designed the experiments; Performed the experiments; Analyzed and interpreted the data; Wrote the paper.

Parmin&Stefanus Muryanto:Contributed reagents, materials, analysis tools or data.

### Funding statement

This work was supported by DIPA Universitas Negeri Semarang (No. 748/UN37.3.1/LT/2016).

### Data availability statement

Data included in article/supplementary material/referenced in article.

### Declaration of interests statement

The authors declare no conflict of interest.

### Additional information

No additional information is available for this paper.

## Appendix A Supplementary data

Ethical Committee Approval Ethical Committee Approval

Research Questionnaire Research Questionnaire

## References

[bib1] Abdurrahman, Widjanarko D., Moeryanto (2019). Implementation of automotive skill competency test through ‘discontinued’ model on vocational school students in Semarang. J. Phys. Conf..

[bib2] Ahmed T. (2016). Labor market outcome for formal vocational education and training in India: safety net and beyond. IIMB Manag. Rev..

[bib3] Åkerstedt T., Axelsson J., Lekander M., Orsini N., Kecklund G. (2014). Do sleep, stress, and illness explain daily variations in fatigue? A prospective study. J. Psychosom. Res..

[bib4] Banks S., Landon L.B., Dorrian J., Waggoner L.B., Centofanti S.A., Roma P.G., Van Dongen H.P.A. (2019). Effects of fatigue on teams and their role in 24/7 operations. Sleep Med. Rev..

[bib5] Bol T., van de Werfhorst H.G. (2011). Signals and closure by degrees: the education effect across 15 European countries. Res. Soc. Stratif. Mobil..

[bib6] Chad K.E., Brown J.M.M. (1995). Climatic stress in the workplace. Appl. Ergon..

[bib7] Choi S.J., Jeong J.C., Kim S.N. (2019). Impact of vocational education and training on adult skills and employment: an applied multilevel analysis. Int. J. Educ. Dev..

[bib8] Forster A.G., Bol T. (2018). Vocational education and employment over the life course using a new measure of occupational specificity. Soc. Sci. Res..

[bib9] Gregson M. (2020). Practice: the importance of practitioner research in vocational education. Educ. Sci..

[bib10] Guo D., Wang A. (2020). Is vocational education a good alternative to low-performing students in China. Int. J. Educ. Dev..

[bib11] Heijke H., Meng C., Ris C. (2003). Fitting to the job: the role of generic and vocational competencies in adjustment and performance. Labor Econ..

[bib12] Hidayatno A., Destyanto A.R., Hulu C.A. (2019). Industry 4.0 technology implementation impact to industrial sustainable energy in Indonesia: a model conceptualization. Energy Proc..

[bib13] Hsouna H., Boukhris O., Abdessalem R., Trabelsi K., Ammar A., Shephard R.J., Chtourou H. (2019). Effect of different nap opportunity durations on short-term maximal performance, attention, feelings, muscle soreness, fatigue, stress and sleep. Physiol. Behav..

[bib14] Janssen N. (2003). Fatigue as a predictor of sickness absence: results from the Maastricht cohort study on fatigue at work. Occup. Environ. Med..

[bib15] Johnson M. (2008). Grading in competence-based qualifications – is it desirable and how might it affect validity?. J. Furth. High. Educ..

[bib16] Lerman S.E., Eskin E., Flower D.J., George E.C., Gerson B., Hartenbaum N., Hursh S.R., Moore-Ede M. (2012). Fatigue risk management in the workplace. J. Occup. Environ. Med..

[bib17] Lim J., Yoon J., Kim M. (2020). Analysis of the educational needs related to, and perceptions of the importance of, essential job competencies among science and engineering graduates. Educ. Sci..

[bib18] Loon M., Bartram T. (2007). Job-demand for learning and job-related learning: the mediating effect of job performance improvement initiative. Int. J. Hum. Resour. Dev. Manag..

[bib19] Maragkou K. (2020). Socio-economic inequality and academic match among post-compulsory education participants. Econ. Educ. Rev..

[bib20] Mohapatra P.K.J., Mandal P., Mahanty B. (1992). Dynamic modelling for age distribution and age- based policies in manpower planning. Appl. Math. Model..

[bib21] Muja A., Blommaert L., Gesthuizen M., Wolbers M.H.J. (2019). The vocational impact of educational programs on youth labor market integration. Res. Soc. Stratif. Mobil..

[bib22] Mulder M. (2007). Competence—the essence and use of the concept in ICVT. Eur. Train Eur. J..

[bib23] Neilson J., Dwiartama A., Fold N., Permadi D. (2020). Resource-based industrial policy in an era of global production networks: strategic coupling in the Indonesian cocoa sector. World Dev..

[bib24] Nilsson A. (2010). Vocational education and training—an engine for economic growth and a vehicle for social inclusion?: vocational education and training. Int. J. Train. Dev..

[bib25] Nurhayati M.N., Siti Zawiah M.D., Mahidzal D. (2016). The relationship between work productivity and acute responses at different levels of production standard times. Int. J. Ind. Ergon..

[bib26] Ocampo A.C.G., Reyes M.L., Chen Y., Restubog S.L.D., Chih Y.-Y., Chua-Garcia L., Guan P. (2020). The role of internship participation and conscientiousness in developing career adaptability: a five-wave growth mixture model analysis. J. Vocat. Behav..

[bib27] O’Neill C., Panuwatwanich K. (2013). Proceedings of the 2013 (4th) International Conference on Engineering, Project, and Production Management.

[bib28] Pelders J., Nelson G. (2019). Contributors to fatigue of mine workers in the South African gold and platinum sector. Health Saf. Work.

[bib29] Pema E., Mehay S. (2012). Career effects of occupation-related vocational education: evidence from the military’s internal labor market. Econ. Educ. Rev..

[bib30] Phillips R.O., Kecklund G., Anund A., Sallinen M. (2017). Fatigue in transport: a review of exposure, risks, checks and controls. Transport Rev..

[bib31] Quiroga-Garza M.E., Flores-Marín D.L., Cantú-Hernández R.R., Eraña Rojas I.E., López Cabrera M.V. (2020). Effects of a vocational program on professional orientation. Heliyon.

[bib32] Rahman A. bt A., Hanafi N., Binti M., Mukhtar M. bt I., Ahmad J. bin (2014). Assessment practices for competency based education and training in vocational college, Malaysia. Proc. Soc. Behav. Sci..

[bib33] Safitri D.S., Rusdiana A. (2010). SPE International Conference on Health, Safety and Environment in Oil and Gas Exploration and Production.

[bib34] Salleh K.M., Sulaiman N.L., Mohamad M.M., Sern L.C. (2015). Academia and practitioner perspectives on competencies required for technical and vocational education students in Malaysia: a comparison with the ASTD WLP competency model. Proc. Soc. Behav. Sci..

[bib35] Sánchez Prieto J., Trujillo Torres J.M., Gómez García M., Gómez García G. (2020). Gender and digital teaching competence in dual vocational education and training. Educ. Sci..

[bib36] Shen J., Barbera J., Shapiro C.M. (2006). Distinguishing sleepiness and fatigue: focus on definition and measurement. Sleep Med. Rev..

[bib37] Suharno, Pambudi N.A., Harjanto B. (2020). Vocational education in Indonesia: history, development, opportunities, and challenges. Child. Youth Serv. Rev..

[bib38] Winther E., Achtenhagen F. (2009). Measurement of vocational competencies—a contribution to an international large-scale assessment on vocational education and training. Emp. Res. Vocat. Educ. Train..

[bib39] Winther E., Klotz V.K. (2013). Measurement of vocational competences: an analysis of the structure and reliability of current assessment practices in economic domains. Emp. Res. Vocat. Educ. Train..

[bib40] Xie X., Xie M., Jin H., Cheung S., Huang C.-C. (2020). Financial support and financial well-being for vocational school students in China. Child. Youth Serv. Rev..

