# Supplementary material for: Evaluation on the automotive skill competency test through ‘discontinuity’ model and the competency test management of vocational education school in Central Java, Indonesia
Source: Heliyon. 2022 Feb 2;8(2):e08872. doi: 10.1016/j.heliyon.2022.e08872 (PMC8899695; doi:10.1016/j.heliyon.2022.e08872)
Supplement: Ethical Committee Approval [file mmc1.pdf]

KEMENTERIAN PENDIDIKAN DAN KEBUDAYAAN
UNIVERSITAS NEGERI SEMARANG
LEMBAGA PENELITIAN DAN PENGABDIAN KEPADA MASYARAKAT
Gedung Prof. Retno Sriningsih Satmoko, Kampus Sekaran, Gunungpati, Semarang 50229
Telp/Fax. (024) 33149439 ext. 1081; (024) 8508089
Laman: http://lppm.unnes.ac.id Surat Elektronik: lppm@mail.unnes.ac.id

SURAT KETERANGAN

Nomor: B/3959/UN37.3.1/PG/2020

This is to verify that this article entitled:

"Evaluation on The Automotive Skill Competency Test Through 'Discontinuity' Model and The Competency Test Management of Vocational Education School in Central Java Indonesia."

Author: 1. Dr. Abdurrahman, M.Pd.
2. Prof. Stefanus Muryanto
3. Dr. Parmin, M.Pd.

has been analyzed ethically based on the regulations made and there is no research writing ethical violations found.

Semarang, 16 November 2020
Director of Research and
Community Service UNNES,

Dr. Suwito Eko Pramono, M.Pd.
NIP 195809201985031003
