# Supplementary material for: Evaluation on the automotive skill competency test through ‘discontinuity’ model and the competency test management of vocational education school in Central Java, Indonesia
Source: Heliyon. 2022 Feb 2;8(2):e08872. doi: 10.1016/j.heliyon.2022.e08872 (PMC8899695; doi:10.1016/j.heliyon.2022.e08872)
Supplement: Research Questionnaire [file mmc2.pdf]

Attachment I. Research Instruments

Students Instrument

Students arè asked to mark the column by giving check. They can check in the column with the number range from 1 to 6. Those numbers sequencely show the level of compatibility between the statements and students response. Number one (1) shows that student is strongly disagreeing with the statement and it continues until number six (6) meaning its statement is fully appropriate with their response. The bigger a number gets, the more agreeable the statement is connected to student response.

No	Statement	Very inappropriate				Appropriate		
		1	2	3	4	5	6	
1	Competency test using 'continuity' model causes physical fatigue							
2	Competency test using 'continuity' model causes vision fatigue							
3	Competency test using 'continuity' model causes ignorance at work due to fatigue							
4	Competency test using 'continuity' model causes heavy load due to the unexistence of breaktime							
5	Competency test using 'continuity' model reduces working enthusiasm due to the unexistence of breaktime							
6	Competency test using 'continuity' model causes mind distraction due to fatigue							
7	Competency test using 'continuity' model causes nervousness since when it is over the test equipments is used by the other students soon.							

No	Statement	Very inappropriate				Appropriate		
		1	2	3	4	5	6	
8	Competency test using 'continuity' model causes ignorance at doing task due to fatigue							
9	Competency test using 'continuity' model causes difficulty to control working attitude due to fatigue.							
10	Competency test using 'continuity' model causes backpain due to the unexistence of breaktime							
11	Competency test using 'continuity' model causes breathing problem due to 5 hours frightful working situation							
12	Competency test using 'continuity' model at Task 4 and 5 causes mental and physical fatigue							
13	Competency test using 'continuity' model does not run optimally due to fatigue							
14	Competency test using 'continuity' model is exhausting making the result imperfect.							
15	Competency test using 'continuity' model should be switched by 'discontinuity' model							
16	Competency test using 'discontinuity' model uses breaktime making students still energized							
17	Competency test using 'discontinuity' model avoids stress due to the existence of sufficient breaktime							
18	Competency test using 'discontinuity' model persists working enthusiasm							
19	Competency test using 'discontinuity' model persists working concentration							

No	Statement	Very inappropriate					Appropriate	
		1	2	3	4	5	6	
20	Competency tëst using 'discontinuity' model avoids student from exstensive fatigue.							
21	Competency test using 'discontinuity' model avoids student form menatl fatigue							
22	Competency test using 'discontinuity' model reduces dullness at doing task							
23	Competency test using 'discontinuity' model gives time to relax the muscle to complete the task well							
24	Competency test using 'discontinuity' model gives enough time to calm the mind (meditation)							
25	Competency test using 'discontinuity' model reduces physical effort than 'continuity' model							
26	Competency test using 'discontinuity' model advances the level of fulfilling the task							
27	Competency test using 'discontinuity' model reduces stress level than 'continuity model							
28	Competency test using 'discontinuity' model reduces physical task than 'continuity model'							
29	Competency test using 'discontinuity' model reduces mental task than 'continuity' model							
30	Competency test using 'discontinuity' model is more pleasant than 'continuity' model							

Competency Test Management

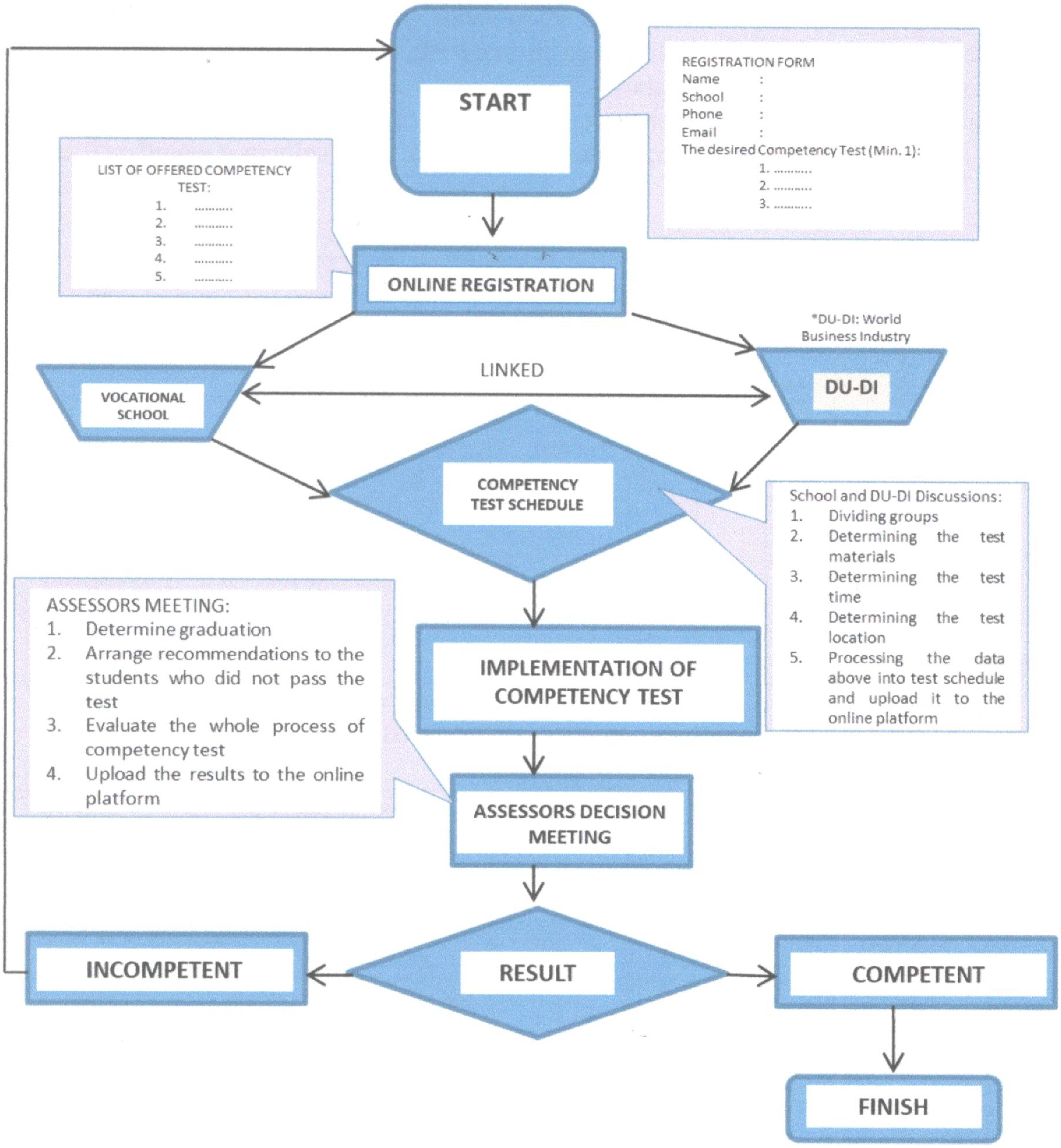

Description:

1. Students fill the registration form by choosing their own test material
2. Students can pick at least one material or maximum five materials
3. After registrants are collected, DUDI and school management conduct subsequent coordination including dividing groups, scheduling the test, determining the test; material, time, place and assessors.
4. Performance of competency test
5. The assessors assemble to decide the passing grade, make the recommendation for students who are not passed through, evaluate overall competency test performance, and upload the test result to the web

Instruments for Head of Department, Head of Automotive Laboratory and Teachers

Head of department, head of automotive laboratory, and teachers are asked to mark the column by giving check. They can check in the column with the number range from 1 to 6. Those numbers sequencely show the level of compatibility between the statements and their response. Number one (1) shows that they are strongly disagreeing with the statement and it continues until number six (6) meaning its statement is fully appropriate with their response. The bigger a number gets, the more agreeable the statement is connected to student response.

No	Statement	Very inappropriate			Appropriate			
		1	2	3	4	5	6	
1	Competency test management model needs more publication due to its complexity							
2	Competency test management model ensures the presence of external assessors (DUDI)							
3	Competency test management model gives freedom to students to be tested based on their readiness							
4	Competency test management model can be carried out anytime							
5	Competency test management model is hard to applied in vocational school							
6	Competency test management model can select the students to do the test since grade 10							

No	Statement	Very inappropriate			Appropriate			
		1	2	3	4	5	6	
7	Competency test management model gives freedom to students to be given the test							
8	Competency test management model is more democratic							

9	Competency test management model gives extra task to students before competency test							
10	Competency test management model produces better competency test quality because it can be assessed by internal and external assessors							
11	Competency test management model should not be performed altogether that might make assessors get tired							
12	Competency test management model is not scheduled as flexible as the usual one							
13	Conventional test model for students who have taken the test causes time wasting while waiting the others done							
14	Competency test management model forces student to do the test based on the schedule							
15	Competency test management model is not appropriate for vocational school							
16	Competency test management model has more benefit than conventional model							
17	Competency test management model needs to be promoted clearly to assessors, students and nead of laboratory							
18	Competency test management model gives a chance to students to redo the test if they do not pass it.							
No	Statement	Very inappropriate				Appropriate		
		1	2	3	4	5	6	
19	Competency test management model need to be realized soon							
20	Competency test management model is more beneficial than conventional one							
21	Competency test management model can be used by many schools to join the test							

22	Competency test management model can train the student to be more toughe since a group could consist students from other vocational schools							
23	Competency test management model is a new way that has never been done in a competency test							
24	Competency test management model is easy to do							
25	Conventional test model should be changed soon by competency test management model							
26	Competency test management model makes students calmer since they communicate frequently to assessors before the test is done							

Respondents may give some suggestions to the Competency test management model as mentioned above. They can add or change the compnonets of the Competency test management model.

Semarang, 17/2 2020

Validator,

Dr. Upik Nurbaiti

196708141991022001
